# FODMAP Profile of Wholegrain Pasta

**DOI:** 10.3390/foods14040667

**Published:** 2025-02-16

**Authors:** Aleksandra M. Torbica, Milorad Miljić, Miloš Radosavljević

**Affiliations:** 1 Institute of Food Technology, University of Novi Sad, Bulevar cara Lazara 1, 21102 Novi Sad, Serbia; milorad.miljic@fins.uns.ac.rs; 2 Faculty of Technology, University of Novi Sad, Bulevar cara Lazara 1, 21102 Novi Sad, Serbia; milosr@tf.uns.ac.rs

**Keywords:** fructooligosaccharides, galactooligosaccharides, fructans, high-FODMAP pasta, IBS, pasta serving size, pasta cooking conditions

## Abstract

Pasta is a staple food consumed worldwide and is made from wholegrain semolina, which is a food rich in dietary fibre, proteins, minerals, vitamins, and bioactive compounds. However, fermentable oligo-, di-, and monosaccharides and polyols (FODMAP), part of soluble dietary fibre in pasta, can trigger/worsen irritable bowel syndrome (IBS) symptoms and increase the prevalence of gastrointestinal disorders. These dietary fibres include lactose, excess fructose relative to glucose, polyols, fructans (mostly fructooligosaccharides), and galactooligosaccharides. Due to a lack of information on the FODMAP profile for pasta, this research conducted a detailed analysis using high-performance anion-exchange chromatography with pulsed amperometric detection to determine the FODMAP compound content in commercially available pasta, with a focus on wholegrain products. The results showed that fructooligosaccharides (FOSs) are the dominant group of FODMAPs, and kestose is the predominant oligosaccharide in all pasta samples both dry (67.1–95.0%) and cooked (27.1–93.9%). Almost all pasta samples are classified as high-FODMAP foods. The degree of reduction in FODMAP compound content during cooking varies between pasta types and is influenced by the wheat type, cooking time, amount of water used for cooking, pasta shapes, and pasta supplementation. In samples of dry pasta, there are statistically significant differences in the results between all samples, while after cooking, there is evident grouping of the results in four clusters. The reduction in FOS content of pasta after cooking was in the range from 30.9% to 84%. Further research should be focused on higher activity of FODMAP degrading enzymes during pasta production process.

## 1. Introduction

Irritable bowel syndrome (IBS) is a chronic gastrointestinal disorder (GID) classified using the ROME IV criteria and indicated by recurrent abdominal pain associated with a change in bowel habits. IBS is very common at present, with a worldwide occurrence of 9% in males and 14% in females [1].

Numerous studies have demonstrated that specific foods elicit IBS symptoms (abdominal pain and bloating) in about 60% of patients, accordingly, the exclusion of foods containing fermentable oligo-, di-, and monosaccharides and polyols (FODMAPs), sucrose, fructose- or gluten-containing foods, and gas-producing foods and prebiotic dietary fibres may have clinical benefits [2]. FODMAPs comprise fermentable oligo-, di-, and monosaccharides and polyols, including lactose, excess fructose relative to glucose, polyols, fructans (mostly fructooligosaccharides), and galactooligosaccharides, with the last two recognized as prebiotics [3]. They are widely distributed in several foods, including simple sugars, fruits and vegetables, wheat, beans, and oats [4]. The accumulation of FODMAPs in the small intestine and their poor absorption result in increased osmotic pressure, which is accompanied by intense fermentation by intestinal microbiota in the colon; this increases the content of gases such as methane, butyrate, and propionate, resulting in abdominal distension and pain [5].

The definition and subsequent classification of dietary fibres (DFs) is difficult and typically based on national regulations. Overall, DFs are defined as non-digestible carbohydrates that are active in the human gut and exhibit scientifically confirmed health benefits. In the USA, Europe, and Canada, oligosaccharides with an average degree of polymerization (DP) of at least 3 are classified as DFs. Thus, oligosaccharides, which include xyloseoligosaccharides (XOSs), fructooligosaccharides (FOSs), and α-galactooligosaccharides (GOSs), are considered DFs [6].

The most abundant FODMAP compounds in grains and grain products are fructans. These linear or branched fructose polymers include short-chain fructans (FOSs) and long-chain fructans (inulin and graminan) [7]. Grains can also possess small quantities of GOSs, mostly raffinose, and stachyose in small or trace quantities. Among other FODMAP compounds present in grains are very small quantities of fructose that do not exceed the glucose content and polyols (mannitol and sorbitol) at 0.01–0.04 g/100 g dry weights [8]. Wheat and rye comprise the main dietary sources of low molecular weight fructans and therefore FODMAPS, with wholegrain products contributing significantly to the intake of both DFs and FODMAPs [9]. Wheat contains several antagonistic compounds/categories that include FODMAPs, gluten, wheat germ agglutinins, and amylase/trypsin inhibitors (ATIs), which can trigger/worsen IBS symptoms. Based on these observations, several restrictive and elimination diets were tested. The gluten-free diet (GFD) has been used to alleviate IBS symptoms with conflicting results. It is used as a last resort and should be avoided for nutritional, social, and economic reasons. Additionally, there is an ongoing discussion in scientific circles regarding the role of wheat components in triggering the symptoms of IBS [2].

The management and treatment of IBS and other FODMAP-related conditions mostly involve dietary changes. Currently, one of the most widely recognized dietary approaches is the implementation of a low-FODMAP diet, which limits the intake of specific fermentable carbohydrates. Reducing FODMAP intake can help alleviate symptoms for many individuals with these specific GIDs. Most dieticians classify the low-FODMAP diet into three stages: restriction, reintroduction, and personalization [3]. The restriction phase focuses on avoiding high-FODMAP foods, which typically results in symptom relief for patients. Although it is effective, the low-FODMAP diet has several restrictions of its own, the most significant of which is the elimination of many common and nutritious foods from the diet. This can lead to nutritional deficiencies, particularly with respect to DFs (which contain important prebiotic components), necessitating careful planning in order to ensure all nutritional needs are met. Furthermore, the low-FODMAP diet can be socially and emotionally challenging due to food choice limitations and potential stigmatization [10]. Overall, the similarities between DF and specific FODMAPs have resulted in criticism of the low-FODMAP diet due to the elimination of healthy DFs. Thus, substituting specific eliminated sources of DFs with beneficial and IBS-suitable DF sources is of the utmost importance. However, it remains challenging to sustain the recommended DF intake of 25–30 g per day [11].

As a staple food, pasta is frequently consumed all over the world. Pasta made from wholegrain semolina is a nutritionally well balanced food that is rich in dietary fibre, vegetable proteins, vitamins, minerals, and functional compounds. Despite the proven benefits of pasta, the growing proportion of the general population that suffers from GID necessitates innovation in pasta making, as wheat and rye pasta, especially wholegrain varieties, are classified as high-FODMAP foods [12].

Due to limited information on the FODMAP content in pasta, this study conducted an in-depth analysis of the content of each FODMAP compound in this scientifically marginalized food category. The literature currently lacks information on the content of specific FODMAP compounds in pasta made from both wholegrain and refined semolina. This study aimed to determine the FODMAP content in both dry and cooked commercially available pasta, with a focus on wholegrain products. In order to fully address the issue of the FODMAP content in pasta, samples made from refined semolina were also included.

## 2. Materials and Methods

### 2.1. Samples

Thirteen samples of pasta commercially available in Serbia were selected. The type, origin, raw materials, and the procedure for cooking the pasta are presented in Table 1.

### 2.2. Chemicals

To quantify the FODMAPs in samples of dry and cooked pasta using high-performance anion-exchange chromatography with pulsed amperometric detection (HPAEC-PAD), extra-pure 50% sodium hydroxide solution (in water) (Fisher Chemical™, Brussels, Belgium) and standards were used. The standards included the following: fructose, galactose, glucose, sucrose, mannitol, sorbitol, 1-kestose, nystose, stachyose hydrate (Sigma-Aldrich, Taufkirchen, Germany), rhamnose monohydrate, xylitol, raffinose pentahydrate (Roth, Lichtentanne, Germany), lactose monohydrate, melibiose, maltitol (Thermo Scientific, Bremen, Germany), verbascose, and 1,1,1-kestopentaose (Megazyme, Bray, Ireland). All carbohydrate reference standards were of >98% purity except for 1,1,1-kestopentaose (80% purity). Ultrapure water with a resistivity of 18.2 MΩ·cm and a total-organic-carbon (TOC) content of <5 µg/kg (ASTM Type I) was used for the preparation of HPAEC-PAD eluents, all standard solutions, and samples; it was obtained from an Adrona Crystal pure water purification system (Adrona SIA, Latvia).

### 2.3. Quantification of FODMAPs Using HPAEC-PAD Method

All samples of dry pasta were ground, and samples of cooked pasta were freeze-dried and ground afterward. The samples were frozen and stored for 24 h at −30 °C and then lyophilized using a ChristALPHA1-2 LDPLUS (Martin Christ, Osterode am Harz, Germany). The lyophilization parameters were as follows: the main drying process was performed at 1000 Pa for 24 h under a condenser temperature of −40 °C and a shelf temperature of room temperature (20–30 °C); the final drying lasted for 24 h at 0.5 Pa under a condenser temperature of −57 °C and at a shelf temperature of room temperature (20–30 °C). Then, 50 mL of ultrapure water was added to 0.5 g of the ground sample. The samples were then placed in a boiling water bath for 10 min. The samples were then cooled to room temperature and centrifuged at 7168 rfc for 10 min at 4 °C. The separated supernatant was frozen at −20 °C (to enable the separation of any fat present, thus avoiding the use of organic solvents). The frozen supernatant was thawed and centrifuged at a speed of 7168 rfc for 5 min at 4 °C. Finally, 5 mL volumes of the prepared sample were transferred into vials for HPAEC-PAD.

The targeted carbohydrates were separated and quantified using a Dionex ICS-6000+ system (Sunnivale, CA, USA) and an electrochemical detector with a gold working electrode and an AgCl reference electrode.

The eluents for the monosaccharides, disaccharides, and polyols were 10 mmol/L NaOH, 200 mmol/L NaOH, and ultrapure water. The separation of the monosaccharides, disaccharides, and polyols was performed on a Thermo Scientific Dionex CarboPac PA20 analytical column (3 × 150 mm) with an appropriate protective column, using isocratic elution according to the method of Weitzhandler et al. [13].

The separation of oligosaccharides (FOSs and GOSs) was carried out using a Thermo Scientific Dionex CarboPac PA200 analytical column (3 × 250 mm) with an appropriate guard column, using gradient elution according to the method of Ispiryan et al. [14]. The eluents used for separation were 200 mmol/L NaOH, 500 mmol/L NaOH, 500 mmol/L NaOAc, and ultrapure water. Separation was performed on both columns at a temperature of 30 °C, while detection was performed at 25 °C.

**Figure 1:**
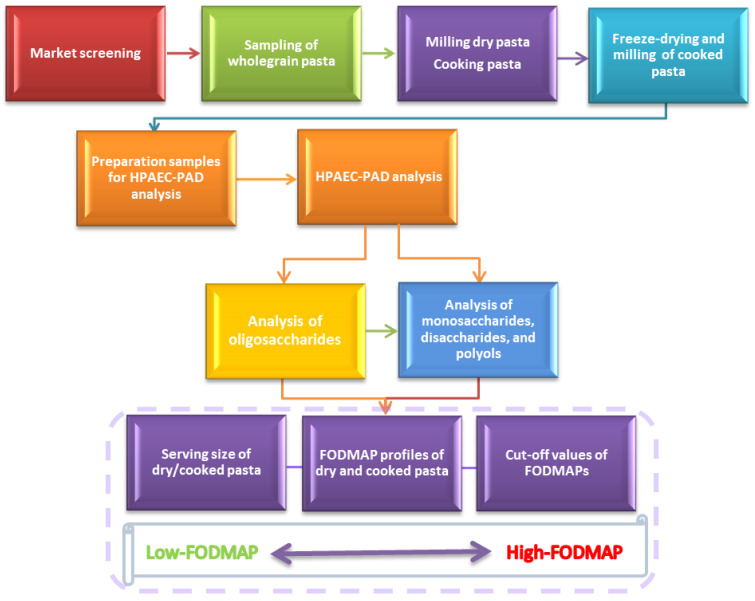
Study’s work flow.

In Figure 1, all steps of the study’s work flow are presented.

## 3. Results and Discussion

### 3.1. FODMAP Compound Reduction During Creation of Cookies and Crackers

The first step toward this goal was a market analysis of wholegrain pasta in order to determine the number of existing products in this category in which to analyze the content of all FODMAP compounds: excess fructose relative to glucose, mannitol, sorbitol, total polyols, lactose, FOS and GOS compounds, and total oligosaccharides, including melibiose (disaccharide), which is considered a FODMAP compound according to recent research [15,16]. Based on the market analysis, the authors observed that there is a limited number of wholegrain pasta on the market. Additionally, there are only a few large manufacturers that produce wholegrain pasta and therefore most of these products are non-standardized pasta produced by small artisan manufacturers. Unlike the majority of studies, the assessment of FODMAP content was performed only on the basis of the HPAC-PAD method and not on the fructan content determined using the enzyme kit because this method is unreliable when measuring low fructan concentrations, such as in wheat [17]. Given that pasta is widely consumed worldwide, it can be concluded that its role in the diets of people with IBS and other gastrointestinal disorders and diseases is underestimated in terms of possible improvements, i.e., reduced-FODMAP wholegrain pasta as an alternative to gluten-free pasta, which is low in FODMAPs but nutritionally inferior to wholegrain pasta.

According to data processed by the International Pasta Organisation (IPO), which promotes the initiative, in the past 25 years, pasta production has increased by almost 85% from 9.1 to almost 17 million tonnes. Today, 40 countries produce over 20,000 tonnes annually, and 52 countries (an increase from 30) consume at least 1 kg per capita each year. The widespread consumption of pasta demonstrates its integration in all cultures and its accessibility to individuals of all social categories [18].

Optimizing the production of low-FODMAP wholegrain pasta could increase pasta intake and overcome existing nutritional deficiencies.

The first step toward this goal was a market analysis of wholegrain pasta in order to determine the number of existing products in this category in which to analyze the content of all FODMAP compounds: excess fructose relative to glucose, mannitol, sorbitol, total polyols, lactose, FOS and GOS compounds, and total oligosaccharides, including the content of melibiose (disaccharide). When using current enzyme kits, the fructan HK method is not appropriate for cereal food samples that have high levels of fructose, glucose, sucrose, or maltose. In experiments with bread samples for which the K-FRUC Assay kit (recommended) was used, the bread contained only 0.2–0.3% fructans rather than 1.0–1.5% dry basis, and these data were confirmed via HPLC [19].

The content of FODMAP compounds was determined in selected samples of wheat pasta available on the market (and one gluten-free pasta made from buckwheat), with the aim of including samples of pasta produced from *Triticum aestivum*, *Triticum durum*, and ancient wheats like spelt and Khorasan. The majority of tested samples were wholegrain pasta because they are crucial in the diet of both healthy individuals and those suffering from GID due to their high TDF content. Pasta samples made from refined semolina (non-wholegrain pasta) were treated as control samples.

The contents of all categories of FODMAP compounds in dry and cooked pasta from the Serbian market are presented in Table 2 and Table 3. 

In order to evaluate the obtained results in terms of defining “low-” or “high-FODMAP” pasta, it was necessary to choose one of the many definitions of serving size for dry and cooked pasta. Due to the complexity and variety of cut-off values for each FODMAP category in terms of the amount per serving and g/100 g of product, the currently available definitions for the European, USA, Canadian, and Australian markets, per regulatory authorities, are summarized in Table 4 and Table 5 [20,21,22,23,24].

**Figure 2:**
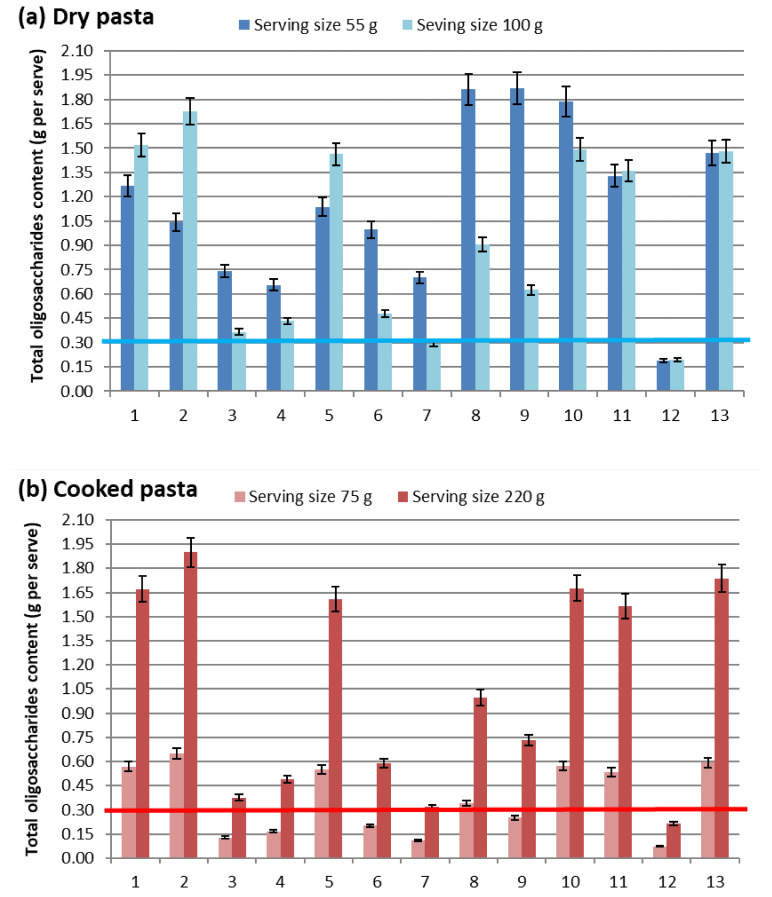
Content of total oligosaccharides in (**a**) dry and (**b**) cooked pasta in g per serving.

In order to assess the impact of serving size on whether the total oligosaccharides exceeded the cut-off value in the samples of dry and cooked pasta, the comparative results are presented in Figure 2. For dry (Figure 2a) and cooked (Figure 2b) pasta, the results were calculated based on serving sizes in Europe (100 g and 220 g, respectively) and Australia (55 g and 140 g, respectively).

By comparing the minimal and maximal serving sizes for dry and cooked pasta (Figure 2a,b), it became evident that an increase in the serving size affects the classification of pasta as a high-FODMAP food. Specifically, the smaller the serving size, the higher the likelihood that the pasta is defined as a low-FODMAP meal. This raises questions about the justification for applying the same cut-off value globally (for a specific category of FODMAP compounds), as serving sizes vary by country, leading to inconsistencies in the objective definition of pasta as a high-FODMAP food.

In order to eliminate the influence of serving size as a criterion on cut-off values, further discussion will focus on the FODMAP content across all categories, expressed as g/100 g “as is”.

### 3.2. Influence of Pasta Cooking Procedure

**Figure 3:**
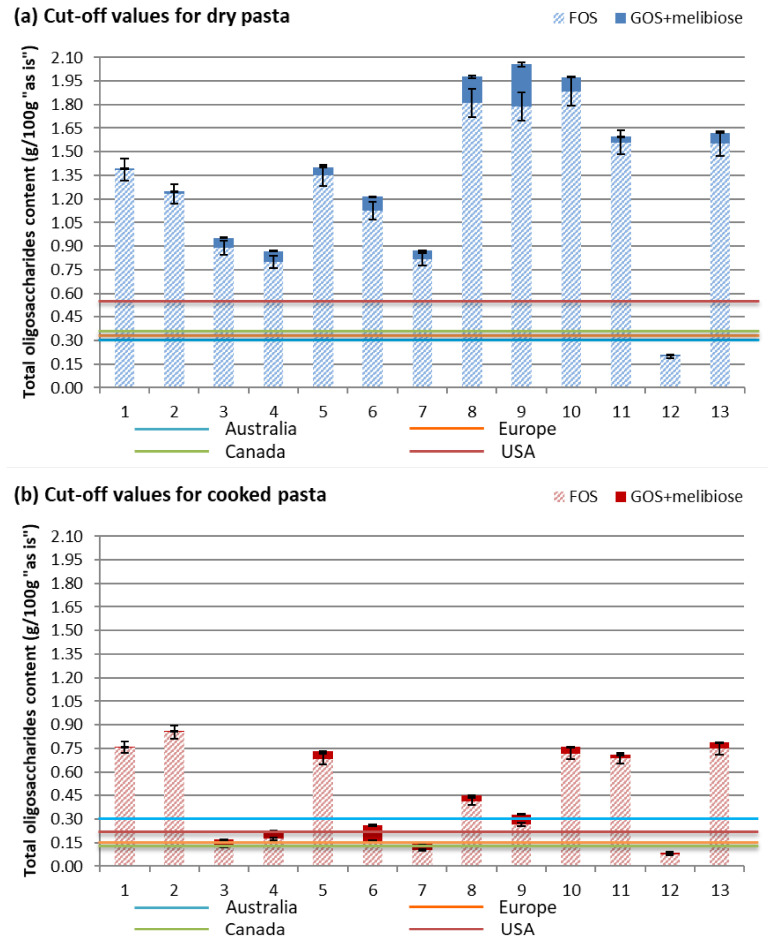
Content of fructoologosaccharides (**a**) and galactoologosaccharides (**b**) in dry and cooked pasta serving.

Analyzing the values obtained through HPAEC-PAD anionic chromatography revealed that the contents of excess fructose (compared to glucose), mannitol, sorbitol, total polyols, and lactose make a negligible contribution to the total FODMAP content in both dry and cooked pasta. Fructans were acknowledged as the major FODMAP compounds present in wheat-based foods, including pasta, as reported by Biesiekierski et al. [25]. The predominant FODMAP compounds are FOSs, but in certain samples, GOSs were decisive in classifying the product as high-FODMAP. FOS and GOS compounds (including melibiose) contributed the most to the total FODMAP content. Nevertheless, FOSs were clearly the dominant group, as shown in the graphical comparison of the contents of these two oligosaccharide groups in Figure 3a,b.

**Figure 4:**
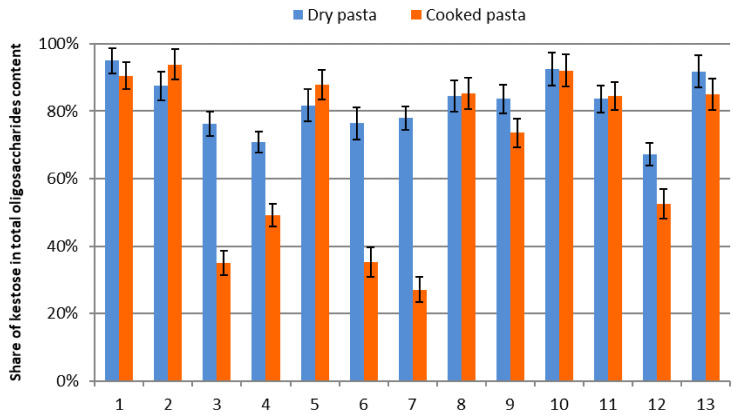
Share of kestose in total oligosaccharides content in dry and cooked pasta.

Kestose is the predominant oligosaccharide within this FOS group (Figure 4). The contents of nystose and kestopentaose are negligible in comparison to kestose, ranging from 0 to 0.085 g/100 g “as is” for nystose and from 0 to 0.152 g/100 g “as is” for kestopentaose.

**Figure 5:**
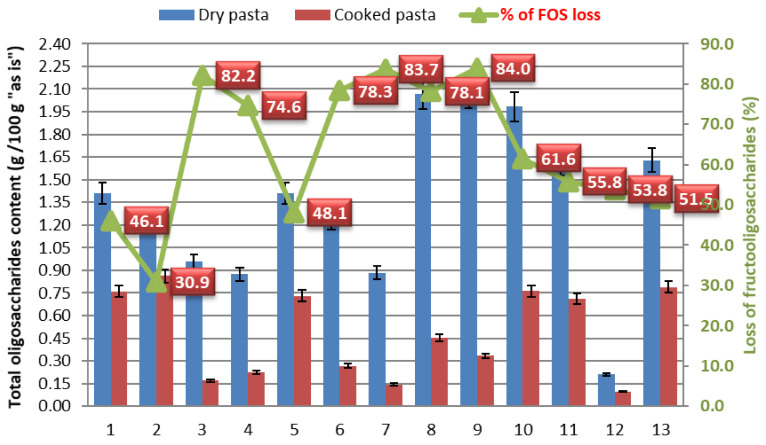
Content of total oligosaccharides in dry and cooked pasta and loss of fructooligosaccharides after cooking.

In order to explain the changes in all FODMAP categories during the pasta-cooking process, the total oligosaccharide content in dry and cooked pasta was considered. Although small amounts of GOSs are present, they can be decisive in the classification of pasta as a high-FODMAP food. A comparison of the total oligosaccharide content in dry and cooked pasta of the same type, expressed as g per 100 g of pasta “as is”, is provided in Figure 5.

The obtained results align with those reported by Ispyrian et al. [8]. Both dried and cooked pasta are classified as high-FODMAP foods due to their high content of FOSs (for both 55 and 140 g per serving). On the other hand, gluten-free pasta is classified as a low-FODMAP food, as was the buckwheat wholegrain pasta (Sample 12) examined in this study.

The expected reduction in FOS and GOS contents after cooking the pasta is a result of the lower dry matter content in cooked pasta compared to dried pasta, as well as the leaching of FODMAP compounds during boiling due to their solubility in water. Fructans are readily solubilized in hot water, leading to the loss of fructan-containing products during cooking [26].

This trend is consistent with the findings of Gélinas et al. who reported that 40–50% of fructans were lost in boiling water while cooking pasta [19]. Similarly, Bustos et al. and Casiraghi et al. observed that cooking pasta in boiling water was very effective at reducing the wheat fructan content, halving the content to 0.8 g of fructans per 100 g of pasta on a dry basis [27,28].

The degree of reduction in FODMAP compound contents during cooking varies between pasta types and is influenced by the wheat type, cooking time, and amount of water used for cooking.

### 3.3. Factors Influencing FODMAP Reduction

#### 3.3.1. Pasta-to-Water Ratio

According to the manufacturers’ instructions, in most cases, 100 g samples of pasta were cooked in 1 L of water. These samples exhibited greater total oligosaccharide (TO) losses during cooking in comparison to samples 10 and 11, for which larger amounts of pasta (200 g and 300 g, respectively) were cooked in 1 l of water.

#### 3.3.2. Duration of Cooking Process

Longer cooking times resulted in greater total losses of oligosaccharides. However, this effect also depended on the shape of the pasta. For example, sample 5 (spaghetti) showed a limited reduction in the total oligosaccharide content even with prolonged cooking, unlike pasta samples with shorter shapes. Gélinas et al. [19] determined that the reduction in oligosaccharides during cooking was not directly related to cooking duration. In this study, however, samples 2, 8, and 13, which were cooked for shorter times, did not exhibit sufficient total oligosaccharide reduction to fall below the cut-off value.

#### 3.3.3. Pasta Shape and Dimensions

Shorter pasta shapes demonstrated greater total oligosaccharide losses during cooking compared to spaghetti, even for samples for which the cooking time was shorter. This could be attributed to the larger specific surface area at the cross-section of shorter pasta shapes.

#### 3.3.4. Raw Material Composition and Supplements

Non-wholegrain pasta exhibited lower total oligosaccharide losses during prolonged cooking because the pasta was supplemented with vitamins C and A. These supplements created an acidic environment during cooking, which is known to hinder the solubility of fructans at low pH levels. However, FOSs were shown to remain relatively stable under acidic conditions coupled with elevated temperature. Courtin et al. [29] observed a maximum of 10% reduction in FOS content when heated to 100 °C under acidic or neutral conditions. In alkaline conditions (pH 11), the FOS content was reduced more drastically (approximately 40%) in comparison to acidic conditions [26].

Since fructans are considered DFs, and their loss during cooking decreases the total DF content of the cooked pasta. This must be considered carefully, particularly for individuals with a healthy GI tract, since the DF content on the label of the uncooked product may differ from that of the cooked product, according to Gélinas et al. [19].

Fructans degrade relatively easily during food processing; thus, making food products that rely on their functional properties could be rather challenging. High temperatures and low pH levels can result in their severe degradation and reduced content. Additionally, processes such as fermentation could lead to significant reductions in the fructan content due to the activity of microbial invertase or inulinase [30].

However, in a recent study, pasta produced from durum flour that was fermented by a pool of selected lactobacilli did not show significant differences in the content of FODMAP compounds compared to a control made with *T. aestivum* flour [2].

## 4. Conclusions

Based on study results, it can be concluded that pasta manufacturers could overcome high-FODMPA content in their products by optimization technological process, e.g., drying pasta at lower temperatures (temperature optimal for FODMAP degrading enzymes) and shaping of short pasta forms. Furthermore, manufacturers should recommend to consumers to cook the pasta in a larger volume of water, at lower temperatures, and under alkaline conditions. Also, there is an evident necessity in the near future to engage medical and food specialists as well as stakeholders to initiate the mandatory addition of information on FODMAP content on the nutrition declaration for all carbohydrate foods. This is of crucial importance for consumers belonging to the IBS and general GID populations.

## Figures and Tables

**Table 1:** The type, origin, raw materials, and the cooking procedure for the pasta.

No.	Producer	Pasta	Ingredients	Nutritional Value/100 g	Water Quantity	Cooking Time
1	Danubius	Wheat spaghetti	Wheat semolina, water, vitamin A (retinyl acetate), vitamin D (cholecalciferol)	Energy Value (kcal) 352, Energy Value (kJ) 1495, Protein (g) 9.16, Carbohydrate (g) 75.46, Sugars (g) 1.4, Fat Total (g) 1.07, Saturated (g) 0.49, Dietary Fibre (g) 2.05, Sodium (g) < 0.01	100 g pasta in 1 L boiling water	9 min
2	Sentella	Wheat penne	Wheat semolina, water	Energy Value (kcal) 337.8, Energy Value (kJ) 1434.2, Protein (g) 9.72, Carbohydrate (g) 72.03, Sugars (g) 1.20, Fat Total (g) 1.20, Saturated (g) 0.18, Dietary Fibre (g) 1.8, Sodium (g) 0.19	100 g pasta in 1 L boiling water	5 min + 2 min *
3	Danubius	Penne rigate durum	Durum wheat semolina, water	Energy Value (kcal) 345, Energy Value (kJ) 1463, Protein (g) 12.11, Carbohydrate (g) 69.22, Sugars (g) 13.7, Fat Total (g) 1.62, Saturated (g) 0.54, Dietary Fibre (g) 2.58, Sodium (g) < 0.01	100 g pasta in 1 L boiling water	9 min
4	Maxi	Durum fusilli	Wheat semolina, water	Energy Value (kcal) 345, Energy Value (kJ) 1483, Protein (g) 12.1, Carbohydrate (g) 69.2, Sugars (g) 1.4, Fat Total (g) 1.6, Saturated (g) 0.5, Dietary Fibre (g) 2.6, Sodium (g) < 0.01	100 g pasta in 1 L boiling water	9 min
5	Danubius	Spaghetti wholegrain durum	Durum wheat semolina, wholegrain wheat flour (10%), water	Energy Value (kcal) 344, Energy Value 1455 (kJ), Protein (g) 12.18, Carbohydrate (g) 65.87, Sugars (g) 1.44, Fat Total (g) 1.86, Saturated (g) 0.36, Dietary Fibre (g) 7.36, Sodium (g) < 0.01	100 g pasta in 1 L boiling water	9 min
6	Makaron SZR	Wholegrain fusili	Wholegrain wheat flour, water	Energy Value (kcal) 339.2, Energy Value 1440.7 (kJ), Protein (g) 12.22, Carbohydrate (g) 70.83, Sugars (g) -, Fat Total (g) 0.78, Saturated (g) -, Dietary Fibre (g) -, Sodium (g) -	100 g pasta in 1 L boiling water	5 min
7	Barilla	Spaghetti wholegrain durum	Wholegrain durum wheat flour, water	Energy Value (kcal) 347, Energy Value (kJ) 1466, Protein (g) 13.0, Carbohydrate (g) 64.0, Sugars (g) 3.5, Fat Total (g) 2.5, Saturated (g) 0.5, Dietary Fibre (g) 8.0, Sodium (g) 0.013	100 g pasta in 1 L boiling water	9 min
8	Jevtić	Khorasan Kamut wholegrain fettuccine	Wholegrain Khorasan Kamut flour (100%)	Energy Value (kcal) 341, Energy Value (kJ) 1440, Protein (g) 12.3, Carbohydrate (g) 62.4, Sugars (g) 1.0, Fat Total (g) 2.65, Saturated (g) 0.3, Dietary Fibre (g) 10.5, Sodium (g) < 0.03	100 g pasta in 1 L boiling water	5 min + 5 min *
9	Jevtić	Spelt wholegrain fettuccine	Wholegrain spelt wheat flour (100%)	Energy Value (kcal) 355, Energy Value (kJ) 1500, Protein (g) 14.0, Carbohydrate (g) 85.0, Sugars (g) 0.8, Fat Total (g) 2.84, Saturated (g) 0.43, Dietary Fibre (g) 11.0, Sodium (g) < 0.03	100 g pasta in 1 L boiling water	5 min + 5 min *
10	Interpak 2015 (Moć prirode)	Wholegrain spelt fusili	Wholegrain spelt flour, wheat semolina, special purpose wheat flour, water	Energy Value (kcal) 364.27, Energy Value (kJ) 1528.74, Protein (g) 12.68, Carbohydrate (g) 73.1, Sugars (g) 3.23, Fat Total (g) 2.35, Saturated (g) 0.07, Dietary Fibre (g) 7.16, Sodium (g) 0.132	200 g pasta in 1 L boiling water	10 min
11	Interpak 2015 (Moć prirode)	Wholegrain rye macaroni	Wholegrain rye flour, special purpose wheat flour, water	Energy Value (kcal) 363, Energy Value (kJ) 1550, Protein (g) 12.0, Carbohydrate (g) 73.5, Sugars (g) 3.5, Fat Total (g) 2.6, Saturated (g) 0.7, Dietary Fibre (g) 7.3, Sodium (g) -	300 g pasta in 1 L boiling water	10 min
12	Interpak 2015 (Moć prirode)	Wholegrain buckwheat pipe rigate	Wholegrain buckwheat flour, special purpose wheat flour, water	Energy Value (kcal) 365, Energy Value (kJ) 1548, Protein (g) 12.7, Carbohydrate (g) 72.5, Sugars (g) 3.4, Fat Total (g) 2.7, Saturated (g) 0.6, Dietary Fibre (g) 7.4, Sodium (g) -	200 g pasta in 1 L boiling water	10 min
13	Tanana testenina	100% wholegrain spelt tagliatelle	Wholegrain spelt flour, water	Energy Value (kcal) 380.7, Energy Value (kJ) 1613.15, Protein (g) 13.14, Carbohydrate (g) 73.35, Sugars (g) 2.99, Fat Total (g) 3.86, Saturated (g) 1.13, Dietary Fibre (g) 7.9, Sodium (g) 0.16	100 g pasta in 1 L boiling water	4 min

* The time the samples were left, after the boiling step, in hot water away from the heating source.

**Table 2:** Content of different categories of FODMAP compounds in dry pasta from Serbian market (g/100 g “as is”) *.

Samples	Monosaccharides		Disaccharides		Polyols				Fructooligosaccharides			Galactooligosaccharides		
	Glucose	Fructose	Lactose	Melibiose	Xylitol	Sorbitol	Mannitol	Maltitol	Kestose	Nystose	Kestopentaose	Raffinose	Stachyose	Verbascose
1.	n.d.	0.049 ± 0.003	0.011 ± 0.001	0.005 ± 0.000	n.d.	n.d.	n.d.	0.097 ± 0.005	1.337 ± 0.067	0.049 ± 0.002	n.d.	n.d.	0.006 ± 0.000	n.d.
2.	0.402 ± 0.020	0.020 ± 0.001	n.d.	n.d.	n.d.	n.d.	n.d.	0.012 ± 0.001	1.093 ± 0.055	0.049 ± 0.002	0.092 ± 0.005	n.d.	0.016 ± 0.002	n.d.
3.	0.011 ± 0.001	0.037 ± 0.002	n.d.	0.005 ± 0.000	n.d.	n.d.	n.d.	0.010 ± 0.001	0.731 ± 0.036	0.036 ± 0.002	0.120 ± 0.006	n.d.	0.066 ± 0.003	n.d.
4.	n.d.	0.014 ± 0.001	n.d.	0.005 ± 0.000	n.d.	n.d.	n.d.	0.015 ± 0.001	0.618 ± 0.031	0.029 ± 0.001	0.152 ± 0.008	n.d.	0.069 ± 0.004	n.d.
5.	0.122 ± 0.007	0.111 ± 0.006	n.d.	0.007 ± 0.000	0.095 ± 0.005	0.031 ± 0.001	0.001 ± 0.000	n.d.	1.151 ± 0.058	0.057 ± 0.003	0.144 ± 0.007	n.d.	0.049 ± 0.003	n.d.
6.	0.109 ± 0.005	0.162 ± 0.008	n.d.	0.020 ± 0.001	0.101 ± 0.005	0.067 ± 0.004	0.004 ± 0.000	n.d.	0.942 ± 0.047	0.033 ± 0.001	0.150 ± 0.007	n.d.	0.089 ± 0.004	n.d.
7.	0.119 ± 0.006	0.132 ± 0.005	n.d.	0.012 ± 0.001	0.135 ± 0.007	0.089 ± 0.004	0.011 ± 0.001	0.009 ± 0.000	0.688 ± 0.034	0.027 ± 0.001	0.102 ± 0.005	n.d.	0.055 ± 0.002	n.d.
8.	0.101 ± 0.005	0.086 ± 0.004	0.083 ± 0.004	0.008 ± 0.000	0.104 ± 0.005	0.101 ± 0.005	0.014 ± 0.001	n.d.	1.749 ± 0.087	0.062 ± 0.003	n.d.	n.d.	0.168 ± 0.008	n.d.
9.	n.d.	0.053 ± 0.003	n.d.	0.022 ± 0.001	n.d.	n.d.	n.d.	0.013 ± 0.001	1.738 ± 0.086	0.051 ± 0.002	n.d.	n.d.	0.267 ± 0.013	n.d.
10.	0.076 ± 0.004	0.013 ± 0.001	n.d.	0.008 ± 0.000	0.081 ± 0.004	0.032 ± 0.001	0.038 ± 0.002	n.d.	1.834 ± 0.092	0.051 ± 0.002	n.d.	n.d.	0.090 ± 0.004	n.d.
11.	0.171 ± 0.008	0.156 ± 0.008	n.d.	0.012 ± 0.001	0.070 ± 0.004	n.d.	0.006 ± 0.000	n.d.	1.344 ± 0.067	0.085 ± 0.004	0.132 ± 0.006	n.d.	0.036 ± 0.001	n.d.
12.	0.112 ± 0.006	0.205 ± 0.011	n.d.	n.d.	0.087 ± 0.005	0.036 ± 0.002	0.094 ± 0.004	0.032 ± 0.001	0.141 ± 0.007	0.058 ± 0.003	n.d.	n.d.	0.011 ± 0.001	n.d.
13.	0.118 ± 0.006	0.104 ± 0.005	n.d.	0.009 ± 0.000	0.156 ± 0.007	0.068 ± 0.003	0.011 ± 0.001	0.009 ± 0.000	1.496 ± 0.075	0.054 ± 0.002	n.d.	n.d.	0.071 ± 0.003	n.d.

* n.d.—not detected.

**Table 3:** Content of different categories of FODMAP compounds in cooked pasta from Serbian market (g/100 g “as is”) *.

Samples	Monosaccharides		Disaccharides		Polyols				Fructooligosaccharides			Galactooligosaccharides		
	Glucose	Fructose	Lactose	Melibiose	Xylitol	Sorbitol	Mannitol	Maltitol	Kestose	Nystose	Kestopentaose	Raffinose	Stachyose	Verbascose
1.	0.012 ± 0.001	0.025 ± 0.001	0.004 ± 0.000	n.d.	n.d.	n.d.	n.d.	n.d.	0.687 ± 0.034	0.059 ± 0.003	0.010 ± 0.000	n.d.	0.003 ± 0.000	n.d.
2.	0.012 ± 0.001	0.017 ± 0.001	n.d.	0.001 ± 0.000	0.006 ± 0.000	n.d.	n.d.	n.d.	0.810 ± 0.040	0.031 ± 0.002	0.012 ± 0.001	n.d.	0.008 ± 0.000	n.d.
3.	0.015 ± 0.001	0.013 ± 0.001	0.001 ± 0.000	0.001 ± 0.000	0.016 ± 0.001	0.009 ± 0.000	n.d.	n.d.	0.060 ± 0.003	0.056 ± 0.002	0.011 ± 0.001	n.d.	0.044 ± 0.002	n.d.
4.	0.011 ± 0.001	0.014 ± 0.001	n.d.	n.d.	0.020 ± 0.001	0.004 ± 0.000	n.d.	n.d.	0.109 ± 0.005	0.059 ± 0.002	0.007 ± 0.000	n.d.	0.048 ± 0.003	n.d.
5.	n.d.	0.012 ± 0.001	n.d.	0.002 ± 0.000	0.041 ± 0.002	0.012 ± 0.001	n.d.	n.d.	0.642 ± 0.032	0.026 ± 0.001	0.011 ± 0.001	n.d.	0.050 ± 0.002	n.d.
6.	n.d.	0.013 ± 0.001	n.d.	0.005 ± 0.000	0.092 ± 0.005	0.032 ± 0.001	n.d.	n.d.	0.094 ± 0.004	0.052 ± 0.002	0.010 ± 0.000	n.d.	0.105 ± 0.004	n.d.
7.	n.d.	0.004 ± 0.000	n.d.	0.003 ± 0.000	0.035 ± 0.002	0.010 ± 0.001	n.d.	n.d.	0.039 ± 0.002	0.050 ± 0.002	0.013 ± 0.001	n.d.	0.040 ± 0.002	n.d.
8.	n.d.	n.d.	n.d.	0.002 ± 0.000	0.026 ± 0.001	0.007 ± 0.000	n.d.	n.d.	0.386 ± 0.019	0.020 ± 0.001	0.007 ± 0.000	n.d.	0.038 ± 0.002	n.d.
9.	0.030 ± 0.001	0.023 ± 0.001	n.d.	0.005 ± 0.000	0.011 ± 0.001	0.002 ± 0.000	0.014 ± 0.001	n.d.	0.245 ± 0.012	n.d.	0.021 ± 0.001	n.d.	0.063 ± 0.003	n.d.
10.	0.057 ± 0.003	0.037 ± 0.002	n.d.	0.003 ± 0.000	0.030 ± 0.001	n.d.	n.d.	n.d.	0.701 ± 0.035	n.d.	0.015 ± 0.001	n.d.	0.042 ± 0.002	n.d.
11.	0.094 ± 0.005	0.079 ± 0.004	0.002 ± 0.000	0.002 ± 0.000	0.046 ± 0.002	n.d.	n.d.	n.d.	0.600 ± 0.031	0.056 ± 0.003	0.032 ± 0.002	n.d.	0.020 ± 0.001	n.d.
12.	0.135 ± 0.007	0.047 ± 0.002	0.004 ± 0.000	0.007 ± 0.000	0.032 ± 0.002	0.032 ± 0.002	n.d.	n.d.	0.051 ± 0.003	0.023 ± 0.001	0.000 ± 0.000	n.d.	0.015 ± 0.001	n.d.
13.	0.003 ± 0.000	0.025 ± 0.001	0.001 ± 0.000	0.001 ± 0.000	0.083 ± 0.004	n.d.	n.d.	n.d.	0.672 ± 0.034	0.026 ± 0.001	0.051 ± 0.003	n.d.	0.038 ± 0.002	n.d.

* n.d.—not detected.

**Table 4:** Cut-off values for FODMAP compounds per serve and per g/100 g “as is” for dry and cooked pasta.

FODMAP		DRY/UN-COOKED PASTA	COOKED PASTA
Cut-Off Values	g per Serve	g/100 g “as Is”	
**Fructose in excess of glucose**			
USA	0.15	0.27	0.11
Canada		0.18	0.07
Australia		/	0.15
Europe		0.17	0.075
**Total oligosaccharides (fructans and galacto-oligosaccharides)**			
USA	0.3	0.55	0.21
Canada		0.35	0.14
Australia		/	0.30
Europe		0.33	0.15
**Total** **polyols**			
USA	0.4 (or 0.2 mannitol or sorbitol)	0.73 (0.36)	0.28 (0.14)
Canada		0.34 (0.37)	0.19 (0.095)
Australia		/	0.4 (0.2)
Europe		0.44 (0.22)	0.2 (0.1)
**Lactose**			
USA	1	1.82	0.71
Canada		1.17	0.46
Australia		/	1
Europe		1.11	0.5

**Table 5:** Serving sizes for FODMAP compounds per serve for dry and cooked pasta.

Serving Size	DRY/UN-COOKED PASTA	COOKED PASTA
USA	55 g	140 g
Canada	85 g	85 g
Australia	/	75–120 g
Europe	80–100 g	180–220 g

## Data Availability

The original contributions presented in the study are included in the article. Further inquiries can be directed to the corresponding author.
